# Accurate measurement of the bond stress between rebar and concrete in reinforced concrete using FBG sensing technology

**DOI:** 10.1038/s41598-024-52555-w

**Published:** 2024-01-24

**Authors:** Murshalin Ahmed, Yukihiro Matsumoto, Rokhyun Yoon, Susumu Takahashi, Yasushi Sanada

**Affiliations:** 1 Department of Architectural Engineering, Osaka University, Osaka, Japan; 2 Department of Architecture and Civil Engineering, Toyohashi University of Technology, Aichi, Japan; 3 Department of Architecture, Daido University, Aichi, Japan

**Keywords:** Engineering, Civil engineering

## Abstract

Recent earthquakes in several developing countries have shown that reinforced concrete (RC) buildings with improper structural detailing experience severe damage under seismic motions. Using low-quality construction materials such as brick aggregates, resulting in low-strength concrete, significantly impacts the bond between rebar and concrete. Accurate evaluation of the bond performance of such low-strength concrete is one of the key issues for seismic safety assessment of RC buildings, especially in Bangladesh; thus, the bond performance is usually evaluated through laboratory tests. However, conventional measurements of bond stress based on rebar strains measured by electrical resistance strain gauges are likely to negatively impact the bond behavior/performance because of the reduced total contact area between rebar and concrete as well as the changing rebar surface boundary conditions. Under the above social and academic backgrounds, in this study, a new measurement technique that applies fiber Bragg grating (FBG) sensors embedded in optical fiber to rebar strain measurements is developed, and its effectiveness is investigated to realize more accurate measurements of the bond stress between rebar and concrete. Two 70% scaled RC beam-column joint specimens in which the beam rebar was anchored in a straight manner were constructed with identical detailing, except for the beam rebar strain measuring methods. The specimens were then subjected to cyclic lateral loading until failure. By comparing the experimental data acquired by the above two different devices (the FBG sensors and conventional strain gauges), it was found that the experimental bond strength on the beam rebar based on the strain data measured by the FBG sensors was much higher than that from the data measured using conventional strain gauges. Which negatively impacted the test data on the beam-column joint’s capacity in the specimen applied the conventional measuring method, indicating the necessity of the presented method not only for accurate evaluation of the bond stress between rebar and concrete but also for seismic safety assessments of RC buildings.

## Introduction

Many buildings exist in developing countries potentially contain improper structural details which do not adequately meet requirements and/or recommendations according to the design guidelines^1–5^. As a result, such buildings in several developing countries have suffered from severe damage under sudden earthquakes. Seismic design details for modern reinforced concrete (RC) moment-resisting frame buildings have become quite complicated, making rebar arrangement difficult. Exterior beam-column joints are one of the most congested areas for rebar placement in the whole structure^6,7^ because beam rebar needs to be terminated and anchored in the exterior joints. Beam-column joints are a critical link in modern moment-resisting RC structures. Recent seismic events^3–5^ globally have highlighted the vulnerability of the beam-column joints, often resulting in severe damage. In developing countries like Bangladesh, the beam-column joints are even more susceptible to damage/total failure due to the following practical background. Understanding and assessing the seismic resilience of these joints is one of the highest concerns in enhancing the overall structural integrity and resilience of RC structures.

Moreover, in developing countries such as Bangladesh, the construction workers working on construction sites are typically uneducated and often poorly skilled^1,2^. Such workers sometimes do not understand the significance of seismic details in construction and sometimes skip key rebar works. Ensuring proper seismic detailing, such as 90° hooks, 135° hooks or 180° hooks at the end of beam rebar, sometimes becomes a hectic process and is simply ignored by keeping a straight anchorage^8^. This problem became more prominent in Bangladesh after the introduction of deformed rebar in the construction market. On the other hand, cost-effective and efficient design is a prerequisite for any successful building construction. One creative way to reduce construction costs is to use locally available low-cost construction materials. In Bangladesh, stone aggregate is a premium construction material because of its geographical background; thus, in many construction projects, locally available brick aggregates have become an immensely popular low-cost alternative to stone aggregates in concrete production^9,10^. One downside of using brick aggregates is that the strength of concrete made with brick aggregates is lower than that of normal concrete made with stone aggregates. In extreme cases, concrete strengths are found to be as low as  N/mm^2^^2^.

A combination of both low-strength concrete due to the use of brick aggregates and inadequate anchorage without seismic detailing has led to a social problem of existence of seismically low-performance RC buildings in Bangladesh. As a result, accurate evaluation of the bond performance between the low-strength concrete and rebar anchored in a straight manner is one of the key issues for the seismic safety assessment of RC buildings in Bangladesh; thus, the bond performance needs to be evaluated through laboratory tests. However, it is still unclear how to accurately obtain experimental data on the bond stress between rebar and concrete because the conventional measurement of bond stress based on rebar strains measured by electrical resistance strain gauges is likely to negatively impact the bond stress itself, as illustrated in the following.

Conventional electrical resistance strain gauges are vulnerable to water damage by wet concrete during the construction process. Therefore, to measure strain, this type of gauge needs to be laminated by several layers of water-resisting vinyl tapes and wax after installation on rebar. Moreover, to run electricity, a lead wire with cross-sectional dimensions of a few millimeters extends from the gauge and is sometimes placed alongside the rebar. These preliminary processes hamper the bond stress between rebar and concrete by reducing the total available contact area between rebar and concrete as well as changing the rebar surface boundary conditions.

Recently, fiber Bragg grating (FBG) has become a leading optical sensing technology for point strain measurements^11–13^. FBG sensors have been widely used in the field of monitoring critical infrastructures^14,15^. Ling et al. experimentally clarified that the strain measured by an FBG sensor embedded in the test beam showed a linear relationship with the strain measured by the electrical resistance strain gauge mounted on the surface^16^. Chung and Kang compared the strain values measured by FBG-based sensors and electric signal-based sensors and found that the FBG sensing system can be an alternative approach, especially for health monitoring systems for structures suffering from electromagnetic interference^17^. More advantages of optical sensing using FBGs have also been described for conventional electrical strain measurements in other studies^18,19^. While the FBG sensing system offers several advantages it is essential to recognize its limitations as well. FBG requires the control FBG sensor to calibrate the temperature and it increases the sensing costs. FBG sensors have a complex installation process and they are much expensive compared to conventional electrical strain gauges.

In this study, FBG sensors were applied to measure the bond stress between rebar and concrete in an RC exterior beam-column joint to mainly compare the bond strengths measured by FBG sensors and conventional electrical resistance strain gauges and to clarify how to obtain reliable test data. Experimental tests were performed on two commonly detailed RC exterior beam-column joint specimens representing a substandard joint in typical RC buildings in Bangladesh, as mentioned above, while both conventional and FBG strain sensors were installed on the beam rebar, respectively. The effectiveness of the FBG sensing technology is discussed based on the experimental data for the seismic safety assessment of the abovementioned substandard RC buildings with poorly detailed exterior beam-column joints.

The significance of the present study lies in investigating the impact of conventional strain gauges in measuring bond stress. Furthermore, an alternative approach utilizing FBG sensing is provided as a potential solution to accurately measure the bond stress. This study offers a novel approach that could overcome the aforementioned shortcomings, thereby enlightening valuable insights to the field of engineering and structural analysis.

## Methodology

**Figure 1:**
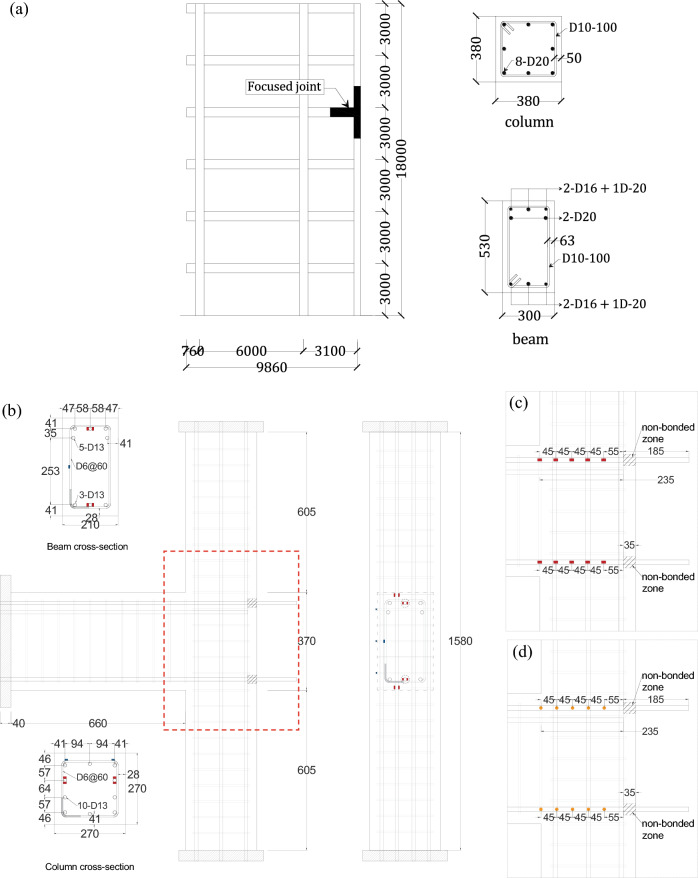
(**a**) Reference building; (**b**) Specimen details; (**c**) Specimen—1 installed conventional electrical resistance strain gauges, showing the arrangement of strain gauges with red squares and (**d**) Specimen—2 installed FBG sensors embedded in optical fiber, showing the arrangement of FBG sensors with yellow circles, unit mm.

In common laboratory tests on RC members, the tensile force acting on the reinforcement is indirectly measured based on the strain measured on the reinforcement surface. The strain on the reinforcement is converted to stress by multiplying by the elastic modulus. In the following experimental study, the strain of beam longitudinal reinforcement within the exterior beam-column joint was measured by two different devices of a conventional electrical resistance strain gauge and an FBG sensor embedded in optical fiber, as shown in Fig. 1. Measurement locations were spaced at 45 mm, starting from the beam-column joint face to the end of the rebar. Two sensors were installed at the opposite side of the rebar at each measurement location for both measurement methods.

### Embedment of the FBG sensor

**Figure 2:**
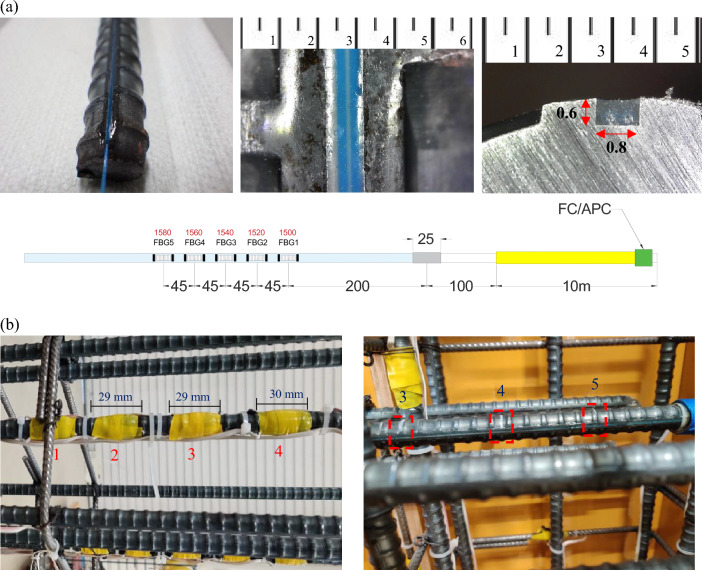
(**a**) FBG sensors embedded in the optical fiber installed in a slit on reinforcement, unit mm; and (**b**) Conventional strain gauge fabrication (left) and FBG-embedded optical fiber fabrication (right). The numbers show each measurement position from the beam-column joint face.

Two narrow 0.8 mm (width) by 0.6 mm (depth) slits were cut into the opposite sides of the subject rebar using a circular machine saw. The slits were cleaned thoroughly, and optical fibers containing the FBG sensors were then embedded in the reinforcement using epoxy. Epoxy was allowed to be set for 24 hours as per the manufacturer’s recommendation. The FBG locations on the optical fiber were predetermined to measure the strain on the beam longitudinal rebar in the beam-column joint region at 45 mm intervals starting from the beam-column joint face, as mentioned above. A total of 5 FBG sensors were installed on each optical fiber. The reflected light wavelength for each individual FBG varied by 20 nm. The reflected light wavelengths were 1580 nm, 1560 nm, 1540 nm, 1520 nm and 1500 nm, as shown in Fig. 2a. The end of the beam longitudinal rebar was extended outside from the column exterior surface to lead the fragile optical fiber outside, as shown in Fig. 1c,d. To ensure the design embedment length of the reinforcement, the bonding between the extended part of the rebar and concrete was removed by wrapping a layer of a Teflon sheet and covering with a PVC pipe, which made no contact between the extended portion of the rebar and the concrete, as shown in Fig. 1c,d. Figure 2a shows the optical fiber with FBG sensors installed in the slit on the reinforcement.

### Installation of conventional electrical resistance strain gauges

Conventional electrical resistance strain gauges were installed at the same locations as the FBG sensors in the beam-column joint region. First, the rebar surface was ground to create a small even surface to place the strain gauge using adhesive. Conventional strain gauges susceptible to water damage were further protected by a layer of wax coating, followed by wrapping with vinyl tape as per the recommendation of the manufacturer. Figure 2b (left) shows the conventional strain gauge installed on the reinforcement.

### Experimental evaluation of the bond stress between rebar and concrete

**Figure 3:**
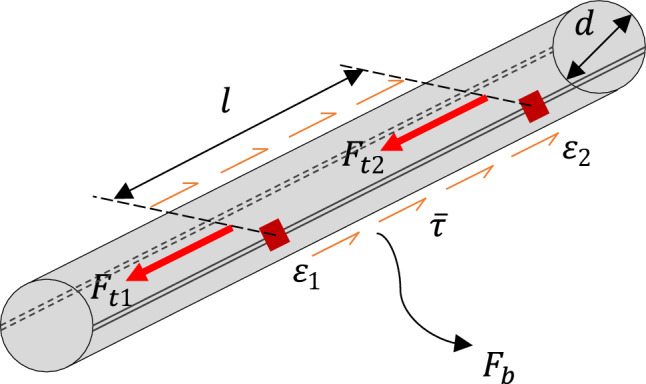
Concept of experimental bond stress evaluation.

Bond stress is commonly evaluated as an averaged value within a predetermined interval length (= 45 mm in this test), as illustrated in Fig. 3. Therefore, the averaged bond stress depends on the interval length. Although the minimal interval length is limited by the dimensions of sensing devices, as shown in Fig. 2b (right), no academic consensus has been established for how the bond stress should be experimentally measured. Here, bond stress was calculated by utilizing an equilibrium between the total bond force at the interval length (${F}_{b}$ by Eq. 1) and the difference in tensile force ($\Delta {F}_{t}$ by Eq. 2) acting on both ends. Figure 3 shows this evaluation concept for the bond stress in a schematic diagram. Consequently, Eq. (3) was derived to experimentally evaluate the average bond stress ($\overline{\tau }$) from the rebar strain outputs.

1 $${F}_{b }=\overline{\tau }\cdot {A}_{s}=\overline{\tau }\cdot \pi \cdot d\cdot l,$$

2 $$\Delta {F}_{t }={F}_{t1}-{F}_{t2}=E\cdot \left({\varepsilon }_{1}-{\varepsilon }_{2}\right)\cdot {A}_{c}=E\cdot \left({\varepsilon }_{1}-{\varepsilon }_{2}\right)\cdot \frac{\pi {d}^{2}}{4},$$

considering $${F}_{b }=\Delta {F}_{t}$$

3 $$\overline{\tau }=\frac{d\cdot E\cdot \left({\varepsilon }_{1}-{\varepsilon }_{2}\right)}{4\cdot l},$$

where ${A}_{s}$ is the surface area, ${A}_{c}$ is the cross-sectional area, and $E$ is Young’s modulus. The other symbols can be found in Fig. 3.

## Experimental program

### Specimen details

Two 70% scaled specimens were constructed representing the exterior beam-column joint of an existing 6-story building located in Dhaka, Bangladesh, as shown in Fig. 1. The specimens were modeled up to the inflection points of the beam and columns, and the member ends were replaced with pin connections. The embedment length of the beam longitudinal rebar was from the column interior face to the column exterior longitudinal rebar of 235 mm with straight anchorage detail, while the rebar was extended outside to lead the optical fiber outside, as mentioned above. Except for the strain measurement devices, both specimens were identical. SD 295^20^ grade rebar with a nominal yield strength of 295 N/mm^2^ was used for both 13 mm (longitudinal rebar) and 6 mm (hoops) diameter rebars.

### Construction details

To represent the realistic conditions of the existing buildings in Bangladesh, both specimens were constructed with brick chips as the coarse aggregate of the concrete. To represent the 70% scaling factor, the bricks were crushed by hand and sieved with dimensions between 5 and 20 mm. The cement-to-sand (fine aggregate)-to-brick chip (coarse aggregate) ratio was 1:2:4, typical for Bangladeshi concrete construction^21^. The water-to-cement ratio was kept at 0.65 with a design concrete strength of 10 N/mm^2^, representing the existing Bangladeshi buildings^1,2,22^. The design yield stress of the reinforcement was 295 N/mm^2^. The specimens were allowed to cure for a minimum of 28 days after casting. The material properties of the concrete and reinforcement as tested before the loading of the specimens are summarized in Table 1.

**Table 1:** Mechanical properties of the construction materials.

Property	Unit	Specimen—1	Specimen—2
Concrete			
Compressive strength	N/mm^2^	12.5	10.8
Youngs modulus	N/mm^2^	8.13 × 10^3^	7.87 × 10^3^
Reinforcement (main bar, 13 mm)			
Yield stress	N/mm^2^	352	
Youngs modulus	N/mm^2^	1.88 × 10^5^	
Reinforcement (hoops, 6 mm)			
Yield stress	N/mm^2^	369	
Youngs modulus	N/mm^2^	1.74 × 10^5^	

### Loading setup

**Figure 4:**
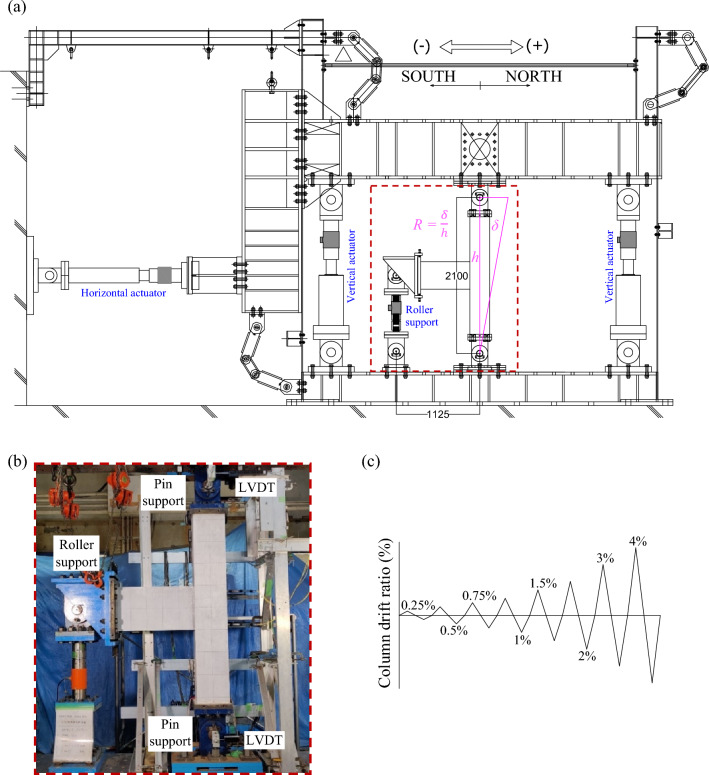
(**a**) Schematic experimental setup, (**b**) specimen during the experiment, and (**c**) lateral load history.

Figure 4a shows the experimental setup. The top and bottom of the column were connected via pins to the loading and foundation stiff steel beams, respectively, and the end of the beam was supported by a roller support free to the horizontal direction. Two vertical hydraulic actuators were used to control the axial loading on the specimen, and one horizontal hydraulic actuator was used to apply static lateral cyclic loading. The horizontal hydraulic actuator was installed in a manner to align with the center of the specimen to ensure double curvature bending of the column. A load cell was incorporated into the horizontal roller support at the beam end to measure the shear force on the beam. Figure 4b shows a photo of the specimen during loading. Figure 4c shows the lateral loading program. The lateral loading was controlled by a horizontal drift angle *R* (rad), as shown in Fig. 4a. One cycle of lateral loading was applied for each peak amplitude in both the positive and negative loading directions since the specimens were expected to fail in a brittle manner. In this study, no axial load was applied to either of the specimens to avoid increasing the bond resistance between the beam longitudinal rebar and concrete under axial compressive loading for the conservative evaluation. Both vertical hydraulic actuators were used to maintain a level state of the loading beam while horizontal loading was applied.

## Test results

**Figure 5:**
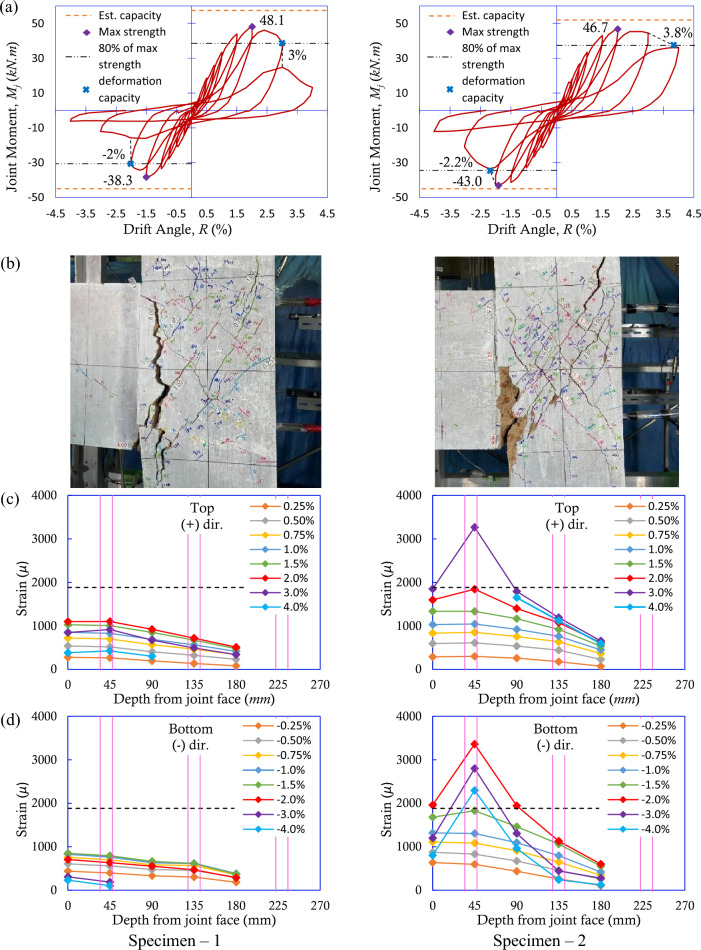
Experimental results: (**a**) joint moment vs. drift angle relationships, showing the estimated capacity by the orange dashed line and 80% of the max capacity by the chain dashed line, (**b**) final damage states, and (**c,d**) strain distributions along the embedded length of the beam longitudinal rebar from the column interior face, showing the yield strain of rebar by the black dashed line.

The test results are summarized in Fig. 5. Figure 5a shows the joint moment–drift angle *R* relationships of both specimens. Here, the joint moment was calculated by multiplying the beam shear force from the load cell incorporated into the horizontal roller support by the beam nodal span length of 1125 mm. The comparison of the damage to both specimens after testing is shown in Fig. 5b. Additionally, the strain distributions of the beam longitudinal rebar on the tension side, namely, the top and bottom rebar in the positive and negative loading directions, respectively, from the column interior face are shown in Fig. 5c,d, in which the schematic locations of the column longitudinal rebar are shown in magenta in the background.

### Failure process of Specimen—1 installed conventional electrical resistance strain gauges

Diagonal cracks appeared at the joint at a ± 0.5% drift angle. Shear reinforcement initially yielded at a + 2.0% drift angle. The maximum strength of 48.1 kN.m was observed at a + 2% drift angle for the positive loading direction and − 38.3 kN.m at a − 1.5% drift angle for the negative loading direction, respectively. A sudden drop in load-carrying capacity coinciding with a wide vertical crack opening was observed at the beam-column joint boundary during the cycles to + 3% drift angle in the positive loading direction and − 2% drift angle in the negative direction, respectively, because pullout failure of the beam longitudinal rebar occurred in both loading directions. No yielding of the beam longitudinal rebar was observed from the conventional strain gauges in either loading direction. The deformation capacity, which was obtained at a point where the specimen reached 80% of the maximum strength, was found to be a 3% drift angle in the positive loading direction and a − 2% drift angle in the negative loading direction, respectively.

### Failure process of Specimen—2 installed FBG sensors embedded in optical fiber

Diagonal cracks at the joint appeared at a ± 0.5% drift angle similar to Specimen—1. Shear reinforcement also similarly yielded at a + 2.0% drift angle. Then, however, different behavior and damage began to be observed in Specimen—2. The maximum strength of 46.7 kN.m was observed at a + 2% drift angle for the positive loading direction and − 43.0 kN.m at a − 2% drift angle for the negative loading direction, respectively. In this specimen, the beam longitudinal rebar yielded during the cycle to + 3%/− 2% drift angle in the positive/negative loading direction. In the positive loading direction, joint cracks widened and extended along the column exterior longitudinal rebar, indicating joint shear failure; thus, no steep loss of load-carrying capacity was observed. In contrast, in the negative loading direction, a sudden drop in the load carrying capacity coinciding with the opening of a vertical crack was observed at the beam-column joint boundary during the cycle to a − 3% drift angle, indicating pullout failure of the beam longitudinal rebar. As a result, the deformation capacity was found to be a 3.8% drift angle and a − 2.2% drift angle in the positive and negative loading directions, respectively.

### Estimated capacity

The ultimate strengths of the specimens were estimated by the following design equations. The flexural strength of the column (*M*_*cu*_) and the flexural strength of the beam (*M*_*bu*_) were calculated according to the Japanese Standard for Seismic Evaluation of Existing Reinforced Concrete Buildings^23^ using Eqs. (4) and (5). The ultimate shear strength of the joint (*V*_*ju*_) was calculated according to the Architectural Institute of Japan Design Guidelines for Earthquake Resistant Reinforced Concrete Buildings Based on Inelastic Displacement Concept^24^ using Eq. (6).

4 $${M}_{cu}=0.8{a}_{t}{\sigma }_{y}{D}_{c}+0.5N{D}_{c}\left(1-\frac{N}{b{D}_{c}{F}_{c}}\right) \left(for\, 0\le N\le 0.4b{D}_{c}{F}_{c}\right),$$

5 $${M}_{bu}=0.9{a}_{t}{\sigma }_{y}d,$$

6 $${V}_{ju}=\kappa \phi {F}_{j}{b}_{j}{D}_{j},$$

where ${a}_{t}$ is the gross tensile area of the rebar, ${\sigma }_{y}$ is the yield stress of the rebar, ${D}_{c}$ is the depth of the column, $N$ is the axial force on the column, $b$ is the width of the column, ${F}_{c}$ is the compressive strength of concrete, $d$ is the effective depth of the beam, $\kappa$ is the shape factor of the joint (0.7 for exterior joint), $\phi$ is factor accounting for the presence of orthogonal beams (0.85 for joint without these beams), ${F}_{j}=0.8{F}_{c}^{0.7}$, ${b}_{j}$ is the effective width of the joint, and ${D}_{j}$ is the effective depth of the joint.

The ultimate strength of the members was converted to the nodal moment according to a previous study^25^. Joint capacity was estimated as the minimum of the three nodal moment capacities. Table 2 summarizes the failure mode evaluation by equivalent joint moment at the ultimate strength. The strength calculation process is explained in details for the Specimen—1 in the positive loading direction for reference in the Supplementary Appendix section. In the positive loading direction, the critical failure mode was governed by joint shear failure, and the estimated capacities were 57.4 kN.m and 51.9 kN.m for Specimens 1 and 2, respectively, as shown in Fig. 5a. The difference in the estimated capacity was due to the difference in the concrete strength of the specimens, as shown in Table 1. In the negative loading direction, the critical failure mode was governed by beam flexural failure, and the estimated capacity was 44.9 kN.m for both specimens, as shown in Fig. 5a.

**Table 2:** Summary of the failure mode evaluation by the equivalent joint moment at the ultimate strength.

Specimen	Loading direction	At column flexural strength (kN.m)	At beam flexural strength (kN.m)	At joint shear strength (kN.m)	Estimated ultimate strength (kN.m)	Failure mode
Specimen—1	Positive	104.0	71.5	**57.4**	57.4	Joint shear failure
	Negative	100.6	**44.9**	57.4	44.9	Beam yielding
Specimen—2	Positive	103.9	71.5	**51.9**	51.9	Joint shear failure
	Negative	100.6	**44.9**	51.9	44.9	Beam yielding

Governing/minimum strength values are in bold.

## Discussions

In the positive loading direction, it was observed that Specimens 1 and 2 achieved 84% and 90% of their estimated capacity, respectively. Conversely, in the negative loading direction, Specimens 1 and 2 achieved 85% and 96% of their estimated capacity, respectively. Compared with Specimen 2, the deformation capacity of Specimen—1 was 21.1% lower in the positive loading direction and 9.1% lower in the negative loading direction when measured using the conventional measurement method, as shown in Fig. 5a.

Importantly, the number of beam longitudinal rebars varied between the positive and negative loading directions for both specimens, as illustrated in Fig. 1b. Thus, the results were analyzed separately for each loading direction. Strain measurements were taken for one out of five rebars (20%) in the positive loading direction and one out of three rebars (33%) in the negative loading direction for both specimens, as shown in Fig. 1b. The failure modes of both specimens were estimated to be joint shear failure in the positive loading direction and beam flexural failure in the negative loading direction, as shown in Table 2.

In the positive loading direction, Specimen—1 began to exhibit brittle pullout failure of the rebar, as evidenced by the strain gauge readings shown in Fig. 5c, after reaching 84% of the expected joint shear capacity. In contrast, Specimen—2 demonstrated the expected joint shear failure, achieving 90% of the anticipated joint shear strength before the failure occurred. Since the specimens were virtually identical, except for minor variations in the concrete strength and the measuring technique utilized, this result highlights the detrimental effect of utilizing the conventional strain measurement method.

In the negative loading direction, both specimens demonstrated similar failure modes, which involved the pullout of the beam after reaching the maximum strength. Ideally, in the case of flexural failure, the maximum strength is attained when the rebars reach their yielding limit. As seen in Fig. 5d, the rebar subjected to a measurement did not yield for Specimen—1 and only achieved 45% of the yield strain. Assuming that the other undisturbed rebars reached their yielding limit, the gross rebar load resisting capacity would have been reduced to approximately 82%, which explains why Specimen—1 was only able to reach 85% of its estimated maximum capacity. Conversely, the rebar subjected to measurement on Specimen—2 yielded, which explains why Specimen—2 was able to approximately reach its estimated capacity.

The damage patterns shown in Fig. 5b indicate that Specimen—1 experienced more concentrated damage near the beam-column joint boundary in both loading directions. However, for Specimen—2, diagonal cracks were distributed evenly across the joint face in the positive loading direction, while in the negative loading direction, they were similar to Specimen—1. These findings, coupled with the load‒deflection results presented in Fig. 5a, suggest that Specimen—1 experienced a more brittle type failure mode than Specimen—2, where FBG sensing was utilized. The use of the conventional strain measurement method, even though partially, has a clear negative impact on the overall behavior of the specimen.

**Figure 6:**
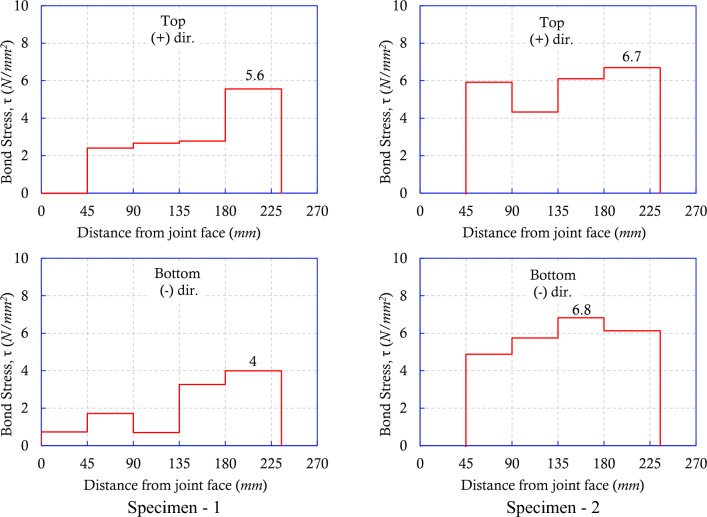
Bond stress of Specimen—1 and Specimen—2.

The bond stress obtained by Eq. (3) utilizing the rebar strain measurements from Fig. 5c,d shows the peak bond strengths in Fig. 6. The conventional measurement method estimated the peak bond strength to be 5.6 N/mm^2^ and 4 N/mm^2^ for the positive and negative loading directions, respectively. Conversely, the FBG sensing measurement method obtained 6.7 N/mm^2^ and 6.8 N/mm^2^ for the positive and negative loading directions, respectively. The conventional measurement method underestimated the critical bond strength capacity by 41.5% in the negative loading direction, where pullout failure was observed due to loss of anchorage of beam longitudinal rebar as a result of the limitation of the bond capacity for both specimens.

The bond strength is a function of compressive strength of concrete^26^. Considering Specimen—1 and Specimen—2, which are nearly identical except for the measurement method and minor variations in concrete strength, the reduced bond strength observed in Specimen—1 is likely to attribute to the diminished net surface contact area between rebar and concrete as a consequence of installing conventional electrical strain gauges.

## Conclusions

This study aimed to evaluate the accuracy and benefits of using the FBG sensing measurement method in comparison to the conventional electrical strain gauge measurement method for determining the bond strength between rebar and concrete in reinforced concrete structures. To achieve this objective, two identical scaled specimens were subjected to lateral cyclic loading until failure while measuring their strain and deformation response using both methods. The major findings of this study are summarized below:

1. The specimen utilizing the conventional measurement method had a lower seismic capacity than the specimens utilizing the FBG sensing method.
2. The measurement method influenced the failure mode of the specimens.
3. The bond strength obtained by the conventional measurement method was 41.5% lower than that obtained by the FBG sensing measurement method.
4. The specimen utilizing the conventional measurement method exhibited a more brittle type of failure compared to the specimens utilizing the FBG sensing method.

### Supplementary Information

Supplementary Information.

## Data Availability

The datasets used and/or analyzed in this study are available from the corresponding author on reasonable request.
