# Supplementary material for: Accurate measurement of the bond stress between rebar and concrete in reinforced concrete using FBG sensing technology
Source: Sci Rep. 2024 Jan 24;14:2119. doi: 10.1038/s41598-024-52555-w (PMC10808180; doi:10.1038/s41598-024-52555-w)
Supplement: Supplementary file 1 — Supplementary Information. [file 41598_2024_52555_MOESM1_ESM.docx]

# Appendix

Strength estimation of the Specimen - 1 in the positive loading direction, as described in Table 2, is shown in detail. Basic dimensions and material properties are explained in Fig. 1 and Table 1.

Flexural strength of the top column at the beam top face is calculated by Eq. (4) as

$M_{cu1}=0.8a_{t}\sigma_{y}D_{c}+0.5ND_{c}\left( 1-\frac{N}{bD_{c}F_{c}} \right) =38.9 kN.m$.

The equivalent joint moment^25^ is ${}_{n}{M_{cu1}}= \frac{L_{c}}{L_{c}-d}\times M_{cu1}=47.2 kN.m$

where $L_{c}$ is the length of column.

Similarly, flexural strength of the bottom column at the beam bottom face is

$M_{cu2}=46.8 kN.m$.

Note: the flexural capacity of top column at beam top face and the bottom column at beam bottom face is different because of the variable axial force acting on the column due to beam end shear force, $V_{b}$.

The equivalent joint moment^25^ is ${}_{n}{M_{cu2}}= \frac{L_{c}}{L_{c}-d}\times M_{cu2}=56.8 kN.m$.

From the above, the total flexural strength of both column is ${}_{n}{M_{cu}}= {}_{n}{M_{cu1}}+{}_{n}{M_{cu2}}=104 kN.m$.

Subsequently, flexural strength of the beam by Eq. (5) is

$$M_{bu}=0.9a_{t}\sigma_{y}d$$

$=63 kN.m$.

The equivalent joint moment^25^ is ${}_{n}{M_{bu}=}\frac{\frac{L_{b}}{2}}{\frac{L_{b}}{2}-\frac{D_{c}}{2}}\times M_{bu}=71.5 kN.m$

where $L_{b}$ is the beam span.

Furthermore, the joint shear strength by Eq. (6) is

$$V_{ju}=\kappa\phi F_{j}b_{j}D_{j}$$

$=157 kN$.

The equivalent joint moment^25^ is ${}_{n}{M_{ju}=}\frac{V_{ju}}{\frac{L_{b}-D_{c}}{L_{b}.j}-\frac{1}{L_{c}}}=57.4 kN.m$

where j is the distance between the compressive/tensile force couple at the beam critical section.

Consequently, the estimated ultimate strength is $M_{u}=\min\left\{ {}_{n}{M_{cu}}, {}_{n}{M_{bu}},{}_{n}{M_{ju}} \right\}=57.4 kN.m$.
